# Scale Estimation and Correction of the Monocular Simultaneous Localization and Mapping (SLAM) Based on Fusion of 1D Laser Range Finder and Vision Data

**DOI:** 10.3390/s18061948

**Published:** 2018-06-15

**Authors:** Zhuang Zhang, Rujin Zhao, Enhai Liu, Kun Yan, Yuebo Ma

**Affiliations:** 1 Institute of Optics and Electronics of Chinese Academy of Sciences, Chengdu 610209, China; zhangzhuang91@126.com (Z.Z.); lehioe@163.com (E.L.); yankunioe@163.com (K.Y.); MYB_IOE@163.com (Y.M.); 2 University of Chinese Academy of Sciences, Beijing 100149, China

**Keywords:** SLAM, sensors fusion, scale estimation, mapping

## Abstract

This article presents a new sensor fusion method for visual simultaneous localization and mapping (SLAM) through integration of a monocular camera and a 1D-laser range finder. Such as a fusion method provides the scale estimation and drift correction and it is not limited by volume, e.g., the stereo camera is constrained by the baseline and overcomes the limited depth range problem associated with SLAM for RGBD cameras. We first present the analytical feasibility for estimating the absolute scale through the fusion of 1D distance information and image information. Next, the analytical derivation of the laser-vision fusion is described in detail based on the local dense reconstruction of the image sequences. We also correct the scale drift of the monocular SLAM using the laser distance information which is independent of the drift error. Finally, application of this approach to both indoor and outdoor scenes is verified by the Technical University of Munich dataset of RGBD and self-collected data. We compare the effects of the scale estimation and drift correction of the proposed method with the SLAM for a monocular camera and a RGBD camera.

## 1. Introduction

Simultaneous localization and mapping (SLAM) may help robots create their own maps while locating themselves in unknown areas by using specific sensors. Recently, there has been a huge scientific interest in a new artificial intelligence research topic, where the robot is capable of sensing the environment. In particular, the monocular SLAM has attracted a widespread attention thanks to its low cost, wide application, and rich information, and has recently made great strides. Similar to more popular cases, e.g., EKF-SLAM [1,2], ORB-SLAM [3,4] and LSD-SLAM [5], a complete monocular SLAM system is formed by the standard extended Kalman filter method, the feature point method or the direct method.

**Figure 1:**
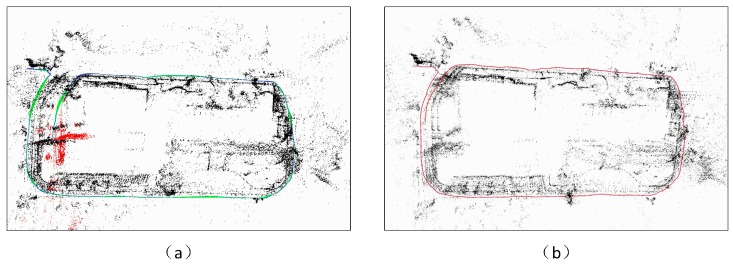
Scale drift. The effect display about ORB-SLAM [1] without detected-loop (**a**) and our proposed fusion LRF and monocular SLAM (**b**). The end of the trajectory should coincide with the starting position.

The monocular SLAM suffers from a particular disadvantage, whereby the camera cannot estimate the absolute scale and therefore, the scale will drift. Figure 1a shows the phenomenon of the scale drift in a very popular ORB-SLAM [1] based on a monocular camera, where no closed loop is detected. In order to solve this issue, we introduce a point distance information into the visual reconstruction to establish a relationship between the visual and real distance. The sensor is assembled by integration of the monocular vision and the 1D laser range finder (LRF). Figure 1b depicts the effect of the scale correction. However, this solution is different from others so that it does not suffer from high hardware costs, exhibits a large detection range, and its accuracy will not be affected by its volume. In the meanwhile, it will not be subject to additional restrictions, so it may be widely used in the construction and positioning process based on the visual 3D motion. In order to achieve this goal, we focus on the following two issues;

- The absolute scale is estimated based on the fusion result of the 1D distance of the LRF and image of the monocular camera.
- Correcting the scale drift of the monocular SLAM using the laser distance information which is independent of the drift error.

This paper is organized as follows: The existing methods related our work is outlined in the following section. Section 3 illustrates the proposed algorithm by mathematical description. In Section 4 and Section 5, we evaluate the solution of the scale estimation and correction by deploying it on the indoor sequences from the Technical University of Munich (TUM) dataset [6] and actual sequences collected by our proposed approach. Finally, conclusions and future are provided in Section 6.

## 2. Related Work

As mentioned earlier, monocular SLAM method has specific weaknesses, where it cannot rely on the structure-from-motion (SFM) technique to restore the absolute scale of the object due to without extra benchmark in the real world [7]. In addition, due to the error accumulation, there is bound to be a drift between the current and the previous reconstructed scales. The drift level will increase in the subsequent process. If it is not corrected in time, the final result will be more serious. Many scholars have put forward constructive proposals for this defect as follows.

It is proposed to reduce the scale drift through uniform optimization of all data. The main idea is to combine the vision SLAM system with the front-end and back-end solutions, the front-end provides initial estimates, and the back-end solves the optimal solution of the overall solution by integrating all the data. For example, Strasdat et al. [8] constructed a monolithic iterative nonlinear optimizer that includes two parts of both bundle-adjustment to densely connected parts of the graph and pose-graph SLAM to weakly connected parts. In [9], Carlone used a closed-form optimization approach called linear approximation for the pose graph optimization (LOAGO). It converts the pose-graph problem and formulate it into a quadratic optimization task by relative orientation representation. On this basis, Dubbelman et al. [10] introduced the COP-SLAM method and extended it to an optimization problem in the 3D scene. These tasks represent significant improvements to the back-end and require high computational consumption. It is difficult to achieve good results for areas that have not yet formed a closed loop. Most importantly, it suffers from lack of estimating the absolute.

In addition, some scholars have studied the use of specific prior information to estimate the absolute scale. The basic idea is to estimate the pose of a well know pattern or physical points in the real world. For this, the inverse EKF method [11,12] is more representative, and its key concept is the direct parametrization of the inverse depth of features relative to the camera locations from which they were first viewed. Currently, a classic approach assumes the camera is located at a fixed height above the ground plane to estimate the absolute scale of the environment [7,13,14]. Duncan [15] estimated the absolute scale and integrated it seamlessly into the beam adjustment by recognition of a prior on the size of the objects, (similar [16,17]). There is also a method for correcting the scale drift based on recognizing similar targets again [18]. However, the aforementioned methods require explicit a priori information and cannot be applied when the scene changes. In order to mitigate these constraints, some scholars have introduced the deep learning method into the visual SLAM [19,20,21]. Based on these predecessors, Laina [22] modeled the ambiguous mapping between the monocular images and the depth maps and realized the depth map of the scene given a single RGB image by a fully convolutional architecture encompassing residual learning. Next, Keisuke’s results, published in 2017 [23], integrated the depth map predicted by the convolutional neural network and the depth map acquired by the monocular SLAM to estimate the absolute scale method. These methods are still in their infancy and have high requirements for computing platforms. In order to get more robust measurements, one require to train in advance through large amounts of data.

To achieve more extensive practicality and reduce the dependence on the scene in the real-time SLAM, some scholars prefer another scale estimation and drift correction scheme to employ additional sensors. Martinelli [24] estimated the absolute scale by merging a monocular camera, three orthogonal accelerometers and three orthogonal gyroscopes, analyzed all the observable modes, and established a fast closed-form solution. The similar work has been before [25,26]. One may also integrate other types of non-visual sensors, e.g., GPS [27,28], and wheeled odometers [29,30,31]. These combinations effectively exploit different measurement properties of multiple sensors, providing more dimensional support for the scale estimation. However, each sensor has a limitation on the scene, and the fusion will further restrict the application scenario.

Other people have explored ways to build the stereo vision. For instance, the widely used stereo-SLAM, which uses the real-scale information contained in the fixed baseline between the two cameras to locate and map [32,33]. The depth estimation and the mapping range of the binocular vision heavily rely on the baseline length and the calibration accuracy between the cameras [34]. On the other hand, with the advent of the RGB-D sensors which capture RGB images along with the per-pixel depth information. For instance, the Real Sense, Kinect and Xtion, rely on the combination of the depth image and the RGB image to realize the purpose of scale estimation and drift correction. Zhang et al. [35] applied the bidirectional Fast Library algorithm and the General Iterative Closest Points (GICP) to improve the accuracy of SLAM. Di and Zhao [36] proposed a method based on extended bundle adjustment with integrated 2D and 3D information on the basis of a new projection model. The SLAM method may be formed based on the RGB-D camera [37,38,39]. However, the depth image resolution of the RGBD cameras is not ideal and the depth of the imaging is limited. For example, Microsoft’s Kinect v2 possesses a detection range of 0.5~4.5 m, so it may only be used in the small-scale scene as indoors.

**Figure 2:**
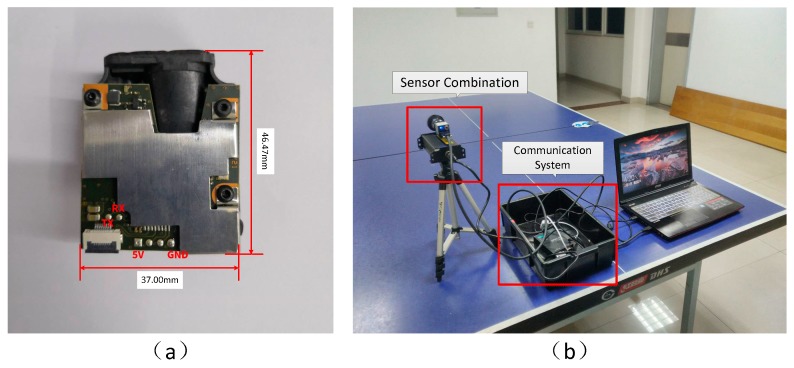
The mono-LRF system assembled via integration of the monocular vision and the LRF. (**a**) Industrial module for 1D laser range finder (LRF); (**b**) The mono-LRF measurement system we built include PC, communication system and sensor combination.

Our goal is to design a SLAM providing a wide range of detection capabilities on the premise of satisfying low power consumption and small volume. Based on lidar odometry, laser ranging requires a high-precision distance sensor with large depth information. The 3D scanning radar in the real-time SLAM suffers from various disadvantages, e.g., huge point cloud data, high power consumption, large size and expensive price. Here we overcome these issues by combining the 1D laser distance information (LRF) and the image information. The small volume of the LRF model is shown as Figure 2a. Figure 2b shows the system with integrated sensors. Afterwards, we show how to use the distance information of one non-feature point in each frame to achieve absolute scale reduction and implement such a new SLAM scheme.

## 3. Method Description

This section systematically elaborates the proposed approach and the initialization including the scale estimation and the drift correction based on laser distance information. The main idea is to establish a correspondence between the one point distances and the matched feature points via quasi-dense reconstruction of the local surface around the laser spot. According to the correspondence, we then estimate the absolute scale and effectively correct the scale drift.

### 3.1. Initialization

The core idea of estimating the absolute scale is to estimate the relative pose of the camera using the SFM [40] and accurately estimate the depth of the space around the laser ranging point. As a result, we establish the absolute scale factor between the initial structure and the real world. However, the sparse point clouds based on the feature reconstruction cannot satisfy this correspondence. An accurate and dense reconstruction of the surface around the laser spot is required. Here, we use a method based on Furukawa’s [41] dense reconstruction to accelerate the calculation of regions.

For a known collection of multi-frame set $\Gamma =\{F^{i}\}$, where $F^{i}$ represents the data of i-th frame, including an image $I^{i}$ and distance of the laser $l_{laser}^{i}$. We select a frame in the middle as reference frame $F^{ref}$ and consider the world coordinate system as the camera coordinate system of the reference frame. Prior to this, we extracted the ORB-feature [42] of image $\left\{I^{i}\right\}$ and completed the matching. Following the SFM [40] method, the estimation of the fundamental matrix or homography is performed on the image sequence [4], and the pose relationship of all frames $\left[R^{i},st^{i}\right]$ (s is the scale factor) are optimized for achieving the bundle adjustment (BA) [43].

#### 3.1.1. Initial Reconstruction

**Figure 3:**
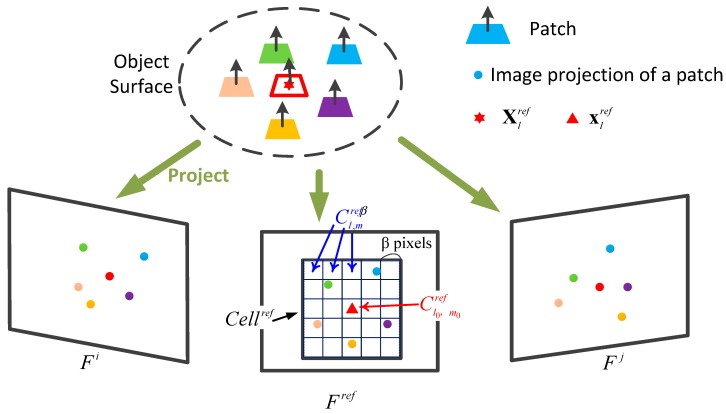
Schematic of the reconstruction, including explanation of parameters and image projections of reconstructed patches in their visible images.

The coordinates of the laser spot is expressed as $X_{l}^{ref}={\left[\begin{array}{cccc} X_{laser}^{ref} & Y_{laser}^{ref} & Z_{laser}^{ref} & 1 \end{array}\right]}^{T}$ in the reference camera coordinate system. It may be calculated from the relative external parameters $\left[R^{laser},t^{laser}\right]$ of the camera and laser that have been previously calibrated, and the distance $l_{laser}^{i}$ from the laser spot. The image projection point may be represented as $x_{l}^{ref}={\left[\begin{array}{ccc} u_{l}^{ref} & v_{l}^{ref} & 1 \end{array}\right]}^{T}$. We then create an $\alpha \times \alpha (pixels^{2})$ area named $Cell^{ref}$, and perform a dense reconstruction of the targets in the $Cell^{ref}$. Next, the area is divided into a grid with β pixels per side of the unit $\left\{C_{l,m}^{ref}\right\}$ (0≤(l,m)<n,α=n•β). In the process of dense reconstruction, we ensure that each patch contains at least one successfully reconstructed patch, or multiple patches (see Figure 3).

Consider the cell $C_{l,m}^{ref}$, in which the feature point $f_{orb}^{ref}$ has been extracted. We then find a set of feature points $\Sigma =\{{f^{\prime }}_{orb}\}$ that matches the points $f_{orb}^{ref}$ of other images, and sort them from near to far according to their distance from the corresponding epipolar line. We construct a candidate patch p for each group of the feature $\left(\begin{array}{cc} f_{orb}^{ref} & \Sigma ({f^{\prime }}_{orb}) \end{array}\right)$, and initialize the patch’s center o(p) and normal v(p). Here, o(p) is achieved by triangulating the matched features, v(p) is the initialization vector of the patch and $O_{ref}$ is the optical center of the reference frame:

(1) $$o(p)\leftarrow \{\begin{array}{ccccc} \mathrm{triangulation} & \mathrm{from} & f_{orb}^{ref} & , & f_{orb}^{i}\begin{array}{cc} ,f_{orb}^{i}\begin{array}{cc} \ldots & \mathrm{and} \end{array} & \left[R^{i},st^{i}\right] \end{array} \end{array}\}$$

(2) $$v(p)=\frac{\vec{o(p)O_{ref}}}{\vec{\left|o(p)O_{ref}\right|}}$$

Next, we use the projection relation to find the visible image of the detected patch p in a specific position of all images. We screen the visibility image $I^{j}$ of the patch p through the local area NCC score and add it to set V(p). The *NCC* score is a function that evaluates the local similarity which will be elaborated in the Appendix A:

(3) $$V(p)=\{\begin{array}{ccc} I^{j}| & I^{j}\in \{F^{j}\},I^{j}\neq I^{ref}, & Ncc(\begin{array}{ccc} p & I^{ref} & I^{j} \end{array})\geq \gamma_{1} \end{array}\}$$

Due to the presence of noise, this initialized value is inaccurate, therefore, we need to subsequently minimize the NCC score to estimate their optimal solution. An evaluation function based on the NCC score may be expressed as:

(4) $$f(\begin{array}{cc} o(p) & v(p) \end{array})=Max\left[\frac{1}{\left|V(p)\right|}\sum_{I_{j}\in (V(p))}Ncc(\begin{array}{ccc} p & I_{ref} & I_{j} \end{array})\right]$$

where o(p) and v(p) are optimization variables to maximize the score $f(\begin{array}{cc} o(p) & v(p) \end{array})$. The positional parameter o(p) is bound by the vector $\vec{o(p)O_{ref}}$ with only one degree of freedom and the direction v(p) possesses two independent degrees of freedom. The Least square method may be used to achieve the optimal solution for the parameters $o(\tilde{p})$ and $v(\tilde{p})$. We may update the visible collection $\tilde{V}(\tilde{p})$ as:

(5) $$\tilde{V}(\tilde{p})=\{\begin{array}{ccc} I_{j}| & I_{j}\in V(p), & Ncc(\begin{array}{ccc} \tilde{p} & I_{ref} & I_{j} \end{array})\geq \gamma_{2} \end{array}\}$$

If $\left|\tilde{V}(\tilde{p})\right|\geq \gamma_{3}$, (usually $\gamma_{3}=3$). Then, it is assumed that the initialization of the patch $\tilde{p}$ is successful, we add it to the patch set $P=\{\tilde{p}\}$.

#### 3.1.2. Patch Proliferation

We next reconstruct patches $p^{\prime }$ for cells $C_{l^{\prime },m^{\prime }}^{ref}$ (the index $\left(l^{\prime },m^{\prime }\right)$) which do not contain an ORB-feature based on the continuity of the object surface. These patches do not satisfy the hypothesis relationship and are introduced by filtering. A dense reconstruction of the area near the target laser spot is achieved. Given an existing patch p and the accordingly cell $C_{l,m}^{ref}$ (the index (l,m)), we look for its neighbor patch $p^{\prime }$ in the direction of the laser projection point $x_{l}^{ref}$. We next describe the index $\left(l_{0},m_{0}\right)$ of the cell $C_{l_{0},m_{0}}^{ref}$ where the laser projection as:

(6) $$\left|l^{\prime }-l\right|+\left|m^{\prime }-m\right|=1$$

(7) $$\left|l^{\prime }-l_{0}\right|+\left|m^{\prime }-m_{0}\right|<=\left|l-l_{0}\right|+\left|m-m_{0}\right|$$

(8) $$P\cup C_{l^{\prime },m^{\prime }}^{ref}=\emptyset$$

One may initialize $p^{\prime }$ by the patch $\tilde{p}$ parameter,

(9) $$o(p^{\prime })\leftarrow \{\mathrm{Intersection}\;\mathrm{of}\;\mathrm{the}\;\mathrm{optical}\;\mathrm{ray}\;\mathrm{through}\;\mathrm{the}\;\mathrm{center}\;\mathrm{of}\;C_{l^{\prime },m^{\prime }}^{ref}\;\mathrm{with}\;\mathrm{plane}\;\mathrm{of}\;\tilde{p}\}$$

(10) $$v(p^{\prime })=n(\tilde{p})$$

(11) $$V(p^{\prime })=\{\begin{array}{ccc} I_{j}| & I_{j}\in \{F^{j}\}, & Ncc(\begin{array}{ccc} p^{\prime } & I_{ref} & I_{j} \end{array})\geq \gamma_{1} \end{array}\}$$

We then take the initial parameters $o(p^{\prime })$ and $v(p^{\prime })$ into the optimization function (4) and solve the optimized optimal solution about $o({\tilde{p}}^{\prime })$ and $v({\tilde{p}}^{\prime })$, for $\left|\tilde{V}({\tilde{p}}^{\prime })\right|\geq \gamma_{3}$ where the expansion is considered successful. It will then be added to the collection P and the collection $N\left(\tilde{p}\right)$ of the neighborhood patches $\tilde{p}$. This process will be repeated until it is expanded for each existing patch around the neighborhood $Near_{l}^{ref}$ of $C_{l_{0},m_{0}}^{ref}$, as

(12) $$Near_{l}^{ref}=\left\{\begin{array}{cc} p^{\prime } & |p\in C_{l_{0},m_{0}}^{ref},p^{\prime }\in C_{l,m}^{ref},\left|l^{\prime }-l_{0}\right|+\left|m^{\prime }-m_{0}\right|=1 \end{array}\right\}$$

Science the reconstruction is based on the assumption of continuity, we filter out the reconstructed patches that do not satisfy this assumption. The patch set U(p) is selected from the adjacent N(p) of the patch *p*, which is not continuous with the patch p. $p^{\prime }(p^{\prime }\in U\left(p\right))$ will make a unified judgment:

(13) $$U\left(p\right)=\left\{\begin{array}{cc} {\tilde{p}}^{\prime } & |\left|\left(o\left(\tilde{p}\right)-o\left({\tilde{p}}^{\prime }\right)\right)\cdot v\left(\tilde{p}\right)\right|+\left|\left(o\left(\tilde{p}\right)-o\left({\tilde{p}}^{\prime }\right)\right)\cdot v\left({\tilde{p}}^{\prime }\right)\right|<\gamma_{4} \end{array}\right\}$$

Among them, $\gamma_{4}$ represents the upper limit of the vertical offset between the two patches. If ${\tilde{p}}^{\prime }$ does not satisfy (13), then it is added to the set denoted by U(p). Two main characteristics of the error patches, combining the experimental experience, are as follows.

The first filter focuses on the outliers that lie outside the real surface. p will be removed from the set P as an error patch when:

(14) $$\left|V\left(\tilde{p}\right)\right|\cdot f\left(\begin{array}{cc} o\left({\tilde{p}}^{\prime }\right), & n\left({\tilde{p}}^{\prime }\right) \end{array}\right)<\sum_{p^{\prime }\in U\left(\tilde{p}\right)}f\left(\begin{array}{cc} o\left({\tilde{p}}^{\prime }\right), & n\left({\tilde{p}}^{\prime }\right) \end{array}\right)$$

Most of the patches that are incorrectly reconstructed are individual patches, connected by few patches as:

(15) $$\frac{\left|N\left(p\right)-U\left(p\right)\right|}{\left|N\left(p\right)\right|}\times 100\%\leq 25\%$$

When the discontinuity patch is discarded, if neighbor $Near_{l}^{ref}$ of the laser cell $C_{l_{0},m_{0}}^{ref}$ still satisfies Equations (14) and (15), then the local dense reconstruction for the reference frame work is completed.

#### 3.1.3. Scale Estimation

We now compare the spatial location $x_{l}^{ref}$ and the reconstructed position $x_{r}^{ref}$ of the p about $C_{l_{0},m_{0}}^{ref}$ to achieve the scale factor.

(16) $$X_{l}^{ref}\leftarrow \{\mathrm{Measurement}\;\mathrm{data}\;\mathrm{from}\;\mathrm{laser}\;\mathrm{range}\;\mathrm{finder}\}$$

(17) $$X_{r}^{ref}\leftarrow \{\mathrm{the}\;\mathrm{intersection}\;\mathrm{of}\;\mathrm{the}\;line(laser)\mathrm{and}\;\mathrm{the}\;plane(p),p\in C_{l_{0},m_{0}}^{ref}\cup p\in Near_{l}^{ref}\}$$

where $X_{l}^{ref}$ is the data from the laser range finder in the system, and $X_{r}^{ref}$ is the point where the viewing ray passing through the projection of the laser point intersecting the plane of the adjacent patch $p_{lrf}$. The scale factor $\lambda_{1}^{ref}$ is the ratio of the true distance $\left|\vec{X_{l}^{ref}O_{lrf}}\right|$ to the reconstructed distance $\left|\vec{X_{r}^{ref}O_{lrf}}\right|$.

(18) $$\lambda_{1}^{ref}=\frac{\left|\vec{X_{l}^{ref}O_{lrf}}\right|}{\left|\vec{X_{r}^{ref}O_{lrf}}\right|}$$

We next select a new frame as the reference frame and repeat the above process to estimate the scale factor $\lambda_{2}^{ref}$, while:

(19) $$\frac{\left|\lambda_{2}^{ref}-\lambda_{1}^{ref}\right|}{\left|\lambda_{2}^{ref}+\lambda_{1}^{ref}\right|}\leq \rho_{0}$$

The scale estimation is judged correct, and the absolute scale $\lambda^{0}$ of the system initialization reads:

(20) $$\lambda^{0}=\frac{1}{2}\left(\lambda_{2}^{ref}+\lambda_{2}^{ref}\right)$$

Based on the scale factor, we restore the real scale $\lambda^{0}$ of the map and adjust the corresponding absolute position of frames. At this point, the system initialization is completed.

### 3.2. Scale Correction

After successful initialization, we estimate the absolute scale. In order to maintain stability of the SLAM system, the scale drift needs to be detected and corrected during the subsequent measurement. Here, we propose the idea of key frame bundling. The key frame selection method is similar to ORB-SLAM [2] and the key frame bundle (KF-Bundle) is the aggregation of multiple key frames combined into a similar initialization $\Gamma^{i}$. We solve the absolute scale of the key frame $\lambda^{i}$ bundling using the same method as in the previous section.

#### 3.2.1. Drift Estimation

Since the scale drift is generated through accumulation of errors, no sudden change occurs in the scale factor relative to the scale of the previous key frame cluster. Therefore, we remove the wrong estimation scale factor via filtering. Once the following two conditions are concurrently satisfied, the scale drift reaches the threshold and the scale update process will be triggered.

(1) The scale of the last three estimates is offset by more than a certain amount relative to the current map scale:
  (21) $$\left|\lambda^{j}-1\right|>\rho_{2},\;j=i-2,i-1,i$$
(2) The scale drift of the current image beam is still expanding:
  (22) $$\left|\lambda^{i}-1\right|>\left(\rho_{2}+\sigma \right)$$

**Figure 4:**
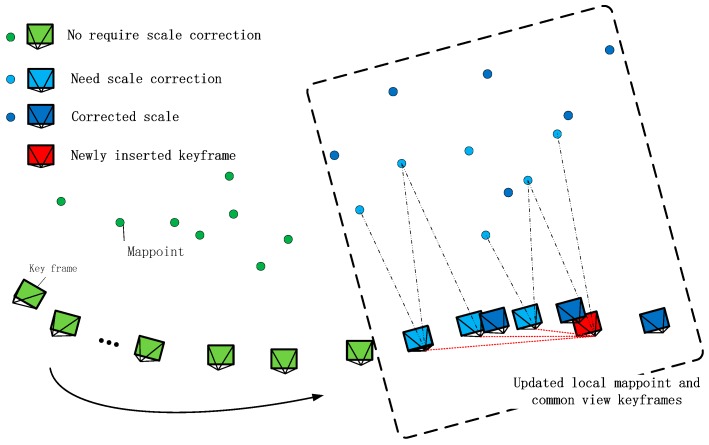
The proposed strategy of the scale correction. The update strategy will be triggered when the drift meets the conditions in Equations (21) and (22).

The value $\left(\begin{array}{cc} \rho_{2} & \sigma \end{array}\right)$ is related to the accuracy of the map and the update frequency of the scale. In the actual experiment, we set it to $\left(\begin{array}{cc} 0.05 & 0.01 \end{array}\right)$ making a trade-off between the computational cost and the positioning accuracy (see Figure 4). The reason is as follows,

- It is not necessary to update each KF cluster since the scale drift is relatively slow and an accurate scale could be followed for a long period.
- Too frequent updating scale takes up computing resources of the system and does not yield corresponding benefits.
- Although we effectively exclude the vast majority of wrong scale estimates, it cannot be rule out there will still be individual errors and frequent updates making the system less stable.

#### 3.2.2. Scale Correction

Once a proper scale estimation is achieved and the updating criteria are met, the correcting flow will be initiated at the next key frame insert. After entering the local optimization process, the key frames having the common view relationship with the new key frame and the map points observed by these key frames will be re-scaled. The detailed process reads:

(1) Inserting a new key frame and enabling the local BA optimization process, the key frames, directly connected to the key frame, will be grouped into a KF-Bundle $\Gamma^{i}$.
(2) The map points observed by the key frames in the $\Gamma^{i}$ will be optimized.
(3) All map points and key frames will be transformed in to the current local coordinate and will be re-scaled by $\lambda^{i}$.
(4) Convert the local coordinates to the world coordinate and these information will be used for the following tracking.

In summary, the 1D laser distance information is used to estimate of the SLAM’s scale parameters and to correct the drift. In this process, we patch the area around the laser spot, and such a scale reconstruction is achieved at low cost.

## 4. TUM Dataset Experiments

Our proposed method requires a distance information provided by a fixed 1D LRF and the image captured by camera. However, in reality, no relevant data set exist for direct use, therefore, we adopt the TUM dataset to this method. The depth information of the center point of the image is then extracted from the depth image to simulate the data of the LRF located at the origin of the camera coordinate system, and directed along the camera’s optical axis. To evaluate the performance of the proposed method, the popular RGB-D dataset (TUM) [4] is used. The TUM dataset exhibits various advantages, i.e., the relative movement of the measurement system is known which allows quantitative evaluation of the proposed method. Moreover, the TUM dataset contains a rich set of scenes and is very challenging because of including fast rotating motion, intense motion blur, and rolling shutter tails. All experiments are performed using a computer equipped with an Intel Core i7-4900MQ @2.80 GHz CPU and 16 Gb RAM. The proposed method run in four main threads, introducing some randomness in the task. For this reason, we report the median from several runs.

### 4.1. Initial Estimation

First, we compared the initialization efficiency and the scale estimation bias under different scenarios in the data set. The number of passed frames before the initialization and the initial scale error is equal to the evaluation standard. The scale error $err_{ini}$ is expressed as:

(23) $$err_{ini}^{M}=\left|\left(d_{i+6}^{M}-d_{i}^{M})-(d_{i+6}^{truth}-d_{i}^{truth}\right)\right|/\left(d_{i+6}^{truth}-d_{i}^{truth}\right)$$

revealing the percentage of the error between the measured distance $d_{i}^{M}$ and the true distance $d_{i}^{truth}$ of the adjacent six frames. To demonstrate the superior performance of our method, we compare it with the RGBD camera of ORB-SLAM [3]. This is a very challenging job since the RGB-D uses a different initialization strategy than ours (iterative closest point (ICP) registration, where there is no limit on the relative rotation), and in the measurement process provides more direct access to the depth of each point information. In order to evaluate the impact of the dense reconstruction on the monocular vision, we introduce the monocular SLAM method as a contrast for the number prior to the initial passed frames.

From Table 1, we may see that for different scenes, similar to the monocular camera method, our method is successfully initialized. This result is related to the SFM [40] initialization method. Since initialization of both the monocular camera method and the proposed method require a rigid condition with parallax between the frames, the initialization success corresponds to the camera movement. Since the camera has started relative motion, we may quickly complete the initialization in fr1/teddy data and others. However, for fr1/xyz due to the beginning of a violently rotation, until above 170 frame of our proposed to complete the initialization. The RGB-D camera method does not require disparity between two frames since it uses the feature point cloud registration algorithm, and may be initialized at every start. For errors, our method is capable of effectively achieving the scale estimation, but not as good as the SLAM for RGBD camera. Since we have greatly reduced the demand for the depth images, we believe that this error range is completely acceptable. In the subsequent measurement, we may eliminate the reconstruction error by filtering the large-scale error estimation through Equations (21) and (22).

### 4.2. Scale Correction

**Figure 5:**
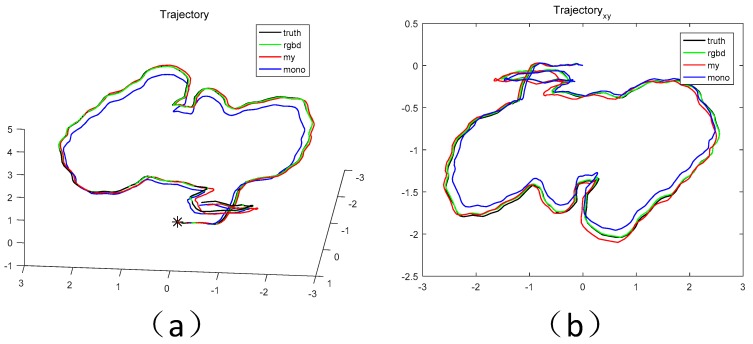
The 3D display of trajectory. (**a**) fr3/office, (**b**) fr3/near.

Let us now evaluate the measurement results achieved from different methods in each scene. In the evaluation process, we mask all methods from the closed-loop optimization threads, in order to verify effectiveness of the proposed scaling update method. We multiply the results of the monocular vision by the true scale of the initial moments due to the lack of the absolute scale in the ORB-SLAM for monocular camera. Figure 5 plots the trajectories corresponding to the measurement results in both stereo and plane scenarios. It may be seen from these figures that in both cases, the proposed method (the red line) is closer to the true value (the black line) than the monocular camera (the blue line), and it is very close to that of the RGBD camera (the green line).

In order to more objectively assess contribution of the proposed method to the scale correction, we refer to the calculation method of the rotation error and mileage error [44]. We set the true value of the rotation matrix and mileage of the camera’s current frame relative to the world coordinate system as $R_{true}^{i}$ and $d_{true}^{i}$, where $R_{true}={\left[\begin{array}{ccc} r_{true}^{1}{}^{\prime } & r_{true}^{2}{}^{\prime } & r_{true}^{3}{}^{\prime } \end{array}\right]}^{T}$, is the total distance of the track, and:

(24) $$\{\begin{array}{c} E_{rot}^{i}(degree)=\max_{k=1}^{3}\{acos(r_{true}^{k}\cdot r^{k})\times 180/\pi \}\\ E_{dist}^{i}(\%)=\left|d_{true}^{i}-d^{i}\right|/\left|d_{true}^{total}\right|\times 100 \end{array}$$

**Figure 6:**
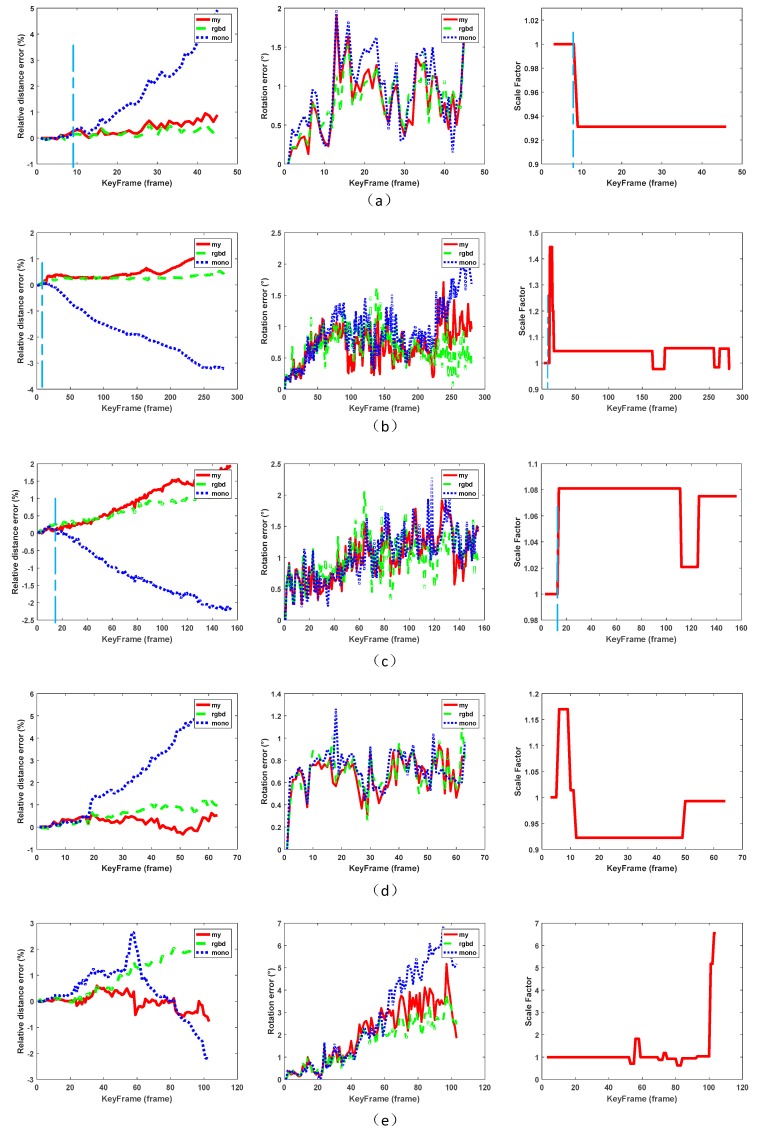
Error assessment results. The horizontal rows of three charts respectively indicate, the relative distance error, the rotation error, and the change curve of the scale estimate. Each row of the data corresponds to a data sequence. (**a**) fr1/xyz; (**b**) fr3/office; (**c**) fr3/near; (**d**) fr3/xyz; (**e**) fr1/teddy.

In this process, we have aligned the results of three methods in time and recorded the changes in the scale factors achieved during the measurement process. Figure 6 plots the result. This figure clearly shows the optimization effect of the proposed method compared to the Monocular SLAM of the relative distance errors. By sudden change of the scale of the monocular vision, our algorithm timely detects the change and maintains the drift within a reasonable range. As shown in Figure 6a, around 9th frame, marked by watery blue, the monocular SLAM rises rapidly. However, the proposed method effectively corrects the scale drift and reduces the distance errors accumulation.

In Figure 6b,d, there are also significant situations like this. A similar effect to RGBD camera is achieved in the final distance estimation error. For the rotation errors, all three methods are based on the corrected matching of the ORB-features. Therefore, similar results are achieved due to the error matches correspond to the image features.

## 5. Self-Collected Dataset Experiments

**Figure 7:**
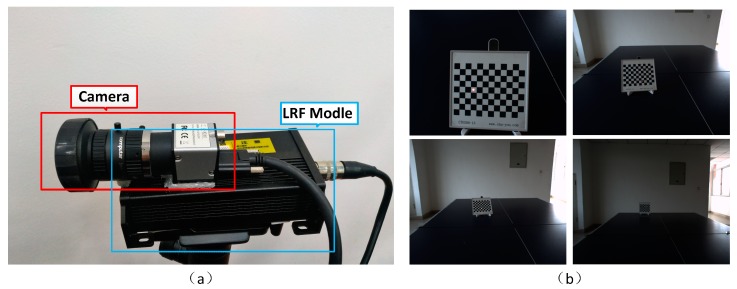
Mono-LRF system and the calibration example. (**a**) Mono-LRF system assembled by the camera and the LRF module; (**b**) Calibration image example for different distances.

Finally, we evaluate the proposed methods on the self-collected data sets. Figure 7a plots the data acquisition system which consists of a camera and a LRF module. We use a “mer131-210” camera with $1280\times 1024\;(pixels^{2})$ resolution and a SDK-100D 1D-LRF module that the measurement range reads 0.3~100 m and the accuracy reads (1+0.5%×distance) (mm). As shown in Figure 7b, we use markers to pre-determine the conversion relationship between the laser coordinate system and the camera coordinate system. Placed on the mobile platform during the trial and evaluated for three different scene.

Since the true value of the pose of the Mono-LRF during the measurement is unknown, we decide to project the estimated trajectory of the camera to Google Maps. Due to the larger scene, the measurement range of the RGBD-camera has been exceeded. Therefore, here we only compare the monocular camera method [2]. We has given the monocular SLAM exactly the same scale information as ours Mono-LRF SLAM within 100 key frames after initialization and shielded closed loop detection to better illustrate the corrective effect on the scale drift. Table 2 lists the measured distances.

### 5.1. Scene 1

**Figure 8:**
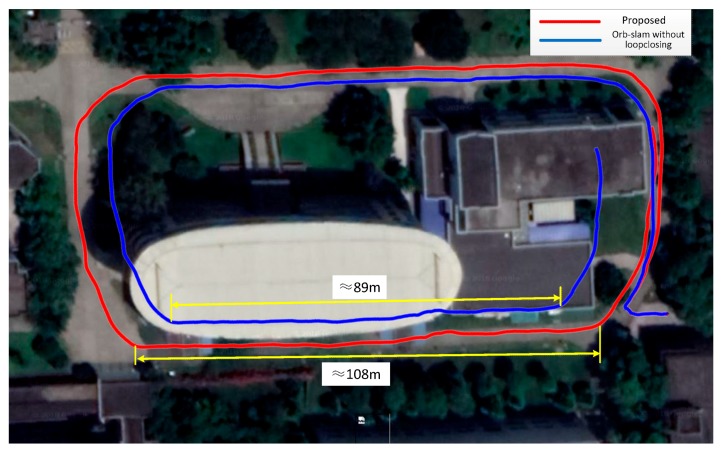
Comparison of the measurement trajectory projections for the proposed method and the Monocular ORB-SLAM method.

Scene 1 is a round of small buildings, Figure 1 shows its reconstruction effect. The GPS has measured distance of 407 m which is close to our result of 405 m. Taking into account the error of the GPS system, this difference is within an acceptable range. Through projection of the trajectories on the Google Maps (Figure 8), we may see the corrective effect of the proposed method on the scale drift.

### 5.2. Scene 2

**Figure 9:**
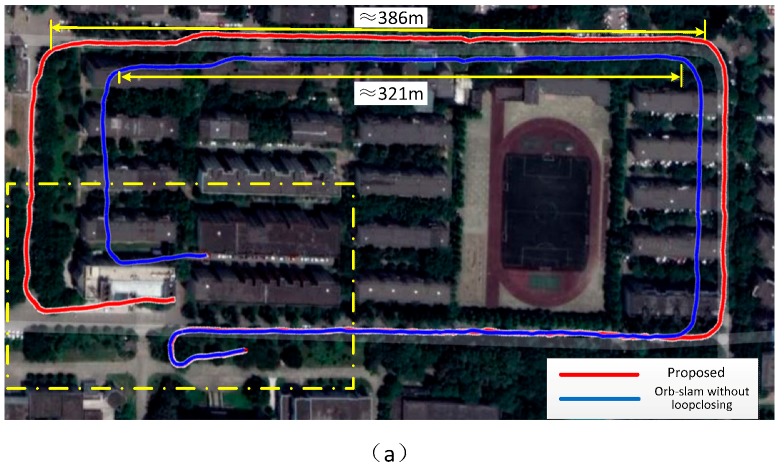

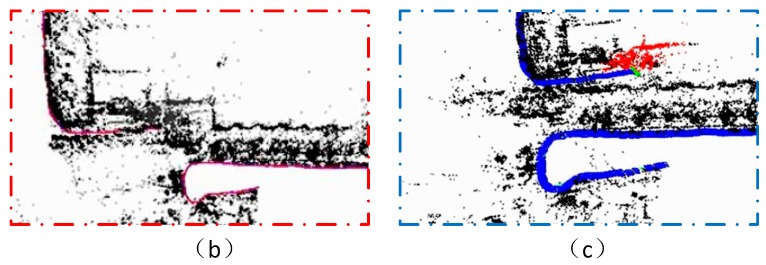
Trajectory in scene 2. (**a**) Comparison of the trajectory projection between the proposed method and the ORB-SLAM for monocular camera. (**b**) Using our method to reconstruct the results at the end. (**c**) Using the monocular SLAM to reconstruct the results at the end.

Scenario 2 is resulted from the measurement of the surround of a larger scene, similar to the result of the scene 1. It is in the course of 1.23 km, considering irregularity of the movement and the influence of the building on the GPS positioning accuracy. The distance measured by the proposed method is slightly smaller than the GPS data. The monocular SLAM is exhibited a larger drift. Figure 9 plots the details and clearly show that our measurements are slightly offset.

### 5.3. Scene 3

**Figure 10:**
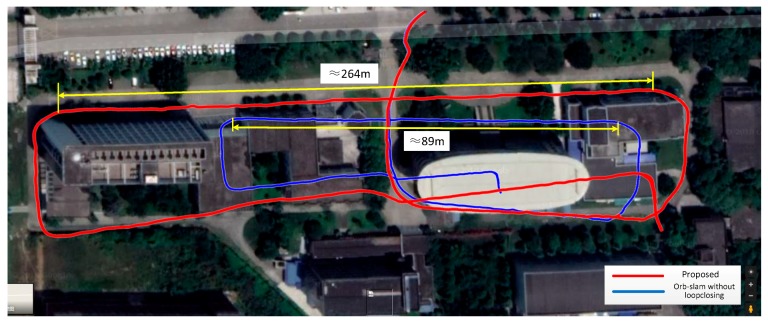
Trajectory in scene 3. The red trajectory is basically the same as the real one, while the blue trajectory has a large scale drift problem.

We next investigate the long-distance scenes that experienced multiple rotations. In the motion, we randomly turn the SLAM system and the results are shown in Figure 10. Increasing the number of the rotations, deviations have occurred in the direction estimation of both algorithms. The scale drift of the monocular ORB-SLAM system has rapidly accumulated, resulting in a large inaccuracy with the real environment. It may be seen from Figure 10 that, the proposed method still maintains a more accurate estimate of the distance traveled than monocular.

## 6. Conclusions

This study introduced a novel SLAM method for achieving the absolute scale estimation and the scaling drift correction through the fusion of 1D laser distance information and monocular vision. We first describe integration of the novel fusion. Such a SLAM method does not have to increase the baseline distance in order to measure a large scene like the binocular vision, and is no longer limited by sensors such as RGBD that are applicable to small-scale measurements. Thanks to the simple structure of the 1D laser ranging system, it is convenient to realize the miniaturization and high integration of the measurement system. The latter part of the paper validates the effectiveness of the proposed method through datasets and collected data. Experimental results using the TUM dataset effectively prove the reliability of our scale estimation, which may be acceptable, compared to the increase of the data in one dimension and the wide spread of the measurement range, although there are still many disparities in comparison with the ORB-SLAM for RGBD cameras. In addition, through the collection of the mobile data, we clearly observed the huge improvement of the proposed method compared to the original ORB-SLAM for monocular cameras.

However, the method presented in this article is not perfect. We have tried to overcome the difficulties and tried to make the method more robust. Errors may occur due to purely rotational motion states and for the lack of texture or extreme discontinuity due to the dependence on the SFM method and the local dense reconstruction. In the future, we will combine other depth estimation methods to further improve the fusion of the laser and vision data. This approach may eventually find applications in the increasingly important robotics industry.

## Figures and Tables

**Table 1:** Evaluation of the initial efficiency and accuracy.

Scene	Data	Proposed Method		RGBD		Monocular
		Number	Err (%)	Number	Err (%)	Number
Testing	fr1/xyz	185	1.30	1	0.11	177
Handheld SLAM	fr3/office	41	5.51	1	0.64	26
No structure	fr3/near	59	4.46	1	0.54	53
Dynamic objects	fr3/xyz	68	4.21	1	0.86	56
3D Reconstruction	fr1/teddy	13	3.66	1	0.61	7

**Table 2:** Measured distance of the movement for different methods.

Scenarios	Frames Number	GPS	Proposed Method	Monocular
1	8871	407 (m)	405 (m)	348 (m)
2	12,787	1.23 (km)	1.17 (km)	954 (m)
3	9921	959 (m)	913 (m)	607 (m)

**Figure A1:**
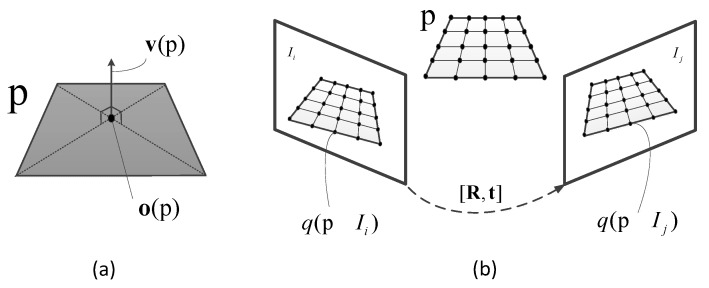
(**a**) A patch is a rectangle with its center and normal denoted as o(p) and v(p), respectively. (**b**) The patch p projected into image and establish a correspondence through bilinear interpolation.

## Appendix A. The Normalized Cross Correlation (NCC) for Patch

NCC is the core metric as in many other algorithms, they apply a robust function to the NCC score to make their photo-consistency robust against outlier signals. For images, the specific expression is as follows:

(A1) $$N=\frac{\sum_{m,n}\left(I_{i}(m,n\right)•I_{j}(m,n))}{\sqrt{\sum_{m,n}I_{i}{\left(m,n\right)}^{2}•\sum_{m,n}I_{j}{\left(m,n\right)}^{2}}}$$

Given a patch p, concretely, a μ×μ grid is overlaid on p and projected into the two images $q(\begin{array}{cc} p & I_{i} \end{array})$, the correlated values being obtained through bilinear interpolation (See Figure A1). We have defined the photometric consistency measure NCC for a patch as a function of its position o(p) and the normal v(p), reconstructing a patch is simply achieved by maximizing the photo-consistency function with respect to those parameters. The mathematical expression is as Equation (A2):

(A2) $$Ncc(\begin{array}{ccc} p & I_{i} & I_{j} \end{array})=\frac{\sum_{m,n}\left(q_{m,n}(\begin{array}{cc} p & I_{i} \end{array}\right)•q_{m,n}(\begin{array}{cc} p & I_{j} \end{array}))}{\sqrt{\sum_{m,n}q_{m,n}{\left(\begin{array}{cc} p & I_{i} \end{array}\right)}^{2}•\sum_{m,n}q_{m,n}{\left(\begin{array}{cc} p & I_{j} \end{array}\right)}^{2}}}$$

## References

[1] Davison A. Real-time simultaneous localization and mapping with a single camera. Proceedings of the Ninth IEEE International Conference on Computer Vision.

[2] Civera J., Davison A.J., Montiel J.M.M. (2012). Inverse Depth Parametrization. Structure from Motion using the Extended Kalman Filter.

[3] Mur-Artal R., Tard S.J.D. (2017). Orb-Slam2: An Open-Source Slam System for Monocular, Stereo, and RGB-D Cameras. IEEE Trans. Robot..

[4] Mur-Artal R., Montiel J.M.M., Tard S.J.D. (2015). Orb-Slam: A Versatile and Accurate Monocular SLAM System. IEEE Trans. Robot..

[5] Engel J., Sch P.S.T., Cremers D. Lsd-Slam: Large-Scale Direct Monocular SLAM. Proceedings of the European Conference on Computer Vision.

[6] Sturm J., Engelhard N., Endres F., Burgard W., Cremers D. A benchmark for the evaluation of RGB-D SLAM systems. Proceedings of the IEEE/RSJ International Conference on Intelligent Robots and Systems.

[7] Zhou D., Dai Y., Li H. Reliable scale estimation and correction for monocular Visual Odometry. Proceedings of the Intelligent Vehicles Symposium.

[8] Strasdat H., Davison A.J., Montiel J.M.M., Konolige K. Double window optimisation for constant time visual SLAM. Proceedings of the International Conference on Computer Vision.

[9] Carlone L., Aragues R., Castellanos J.A., Bona B. (2014). A fast and accurate approximation for planar pose graph optimization. Int. J. Robot. Res..

[10] Dubbelman G., Browning B. (2015). Cop-Slam: Closed-Form Online Pose-Chain Optimization for Visual SLAM. IEEE Trans. Robot..

[11] Civera J., Davison A.J., Montiel J.M.M. Inverse Depth to Depth Conversion for Monocular SLAM. Proceedings of the 2007 IEEE International Conference on Robotics and Automation.

[12] Civera J., Davison A.J., Montiel J.M.M. (2008). Inverse Depth Parametrization for Monocular SLAM. IEEE Trans. Robot..

[13] Scaramuzza D., Fraundorfer F., Pollefeys M., Siegwart R. Absolute scale in structure from motion from a single vehicle mounted camera by exploiting nonholonomic constraints. Proceedings of the IEEE International Conference on Computer Vision.

[14] Song S., Chandraker M. Robust Scale Estimation in Real-Time Monocular SFM for Autonomous Driving. Proceedings of the IEEE Conference on Computer Vision and Pattern Recognition.

[15] Frost D.P., Kähler O., Murray D.W. Object-aware bundle adjustment for correcting monocular scale drift. Proceedings of the IEEE International Conference on Robotics and Automation.

[16] Salas M., Montiel J.M.M. (2016). Real-time monocular object SLAM. Robot. Auton. Syst..

[17] Dame A., Prisacariu V.A., Ren C.Y., Reid I. Dense Reconstruction Using 3D Object Shape Priors. Proceedings of the IEEE Conference on Computer Vision and Pattern Recognition.

[18] Botterill T., Mills S., Green R. (2013). Correcting scale drift by object recognition in single-camera SLAM. IEEE Trans. Cybern..

[19] Liu F., Shen C., Lin G. Deep convolutional neural fields for depth estimation from a single image. Proceedings of the 2015 IEEE Conference on Computer Vision and Pattern Recognition (CVPR).

[20] Eigen D., Fergus R. Predicting Depth, Surface Normals and Semantic Labels with a Common Multi-scale Convolutional Architecture. Proceedings of the 2015 IEEE International Conference on Computer Vision (ICCV).

[21] Gao X., Zhang T. (2017). Unsupervised learning to detect loops using deep neural networks for visual SLAM system. Auton. Robot..

[22] Laina I., Rupprecht C., Belagiannis V., Tombari F., Navab N. Deeper Depth Prediction with Fully Convolutional Residual Networks. Proceedings of the 2016 Fourth International Conference on 3D Vision (3DV).

[23] Tateno K., Tombari F., Laina I., Navab N. CNN-Slam: Real-time dense monocular SLAM with learned depth prediction. Proceedings of the 2017 IEEE Conference on Computer Vision and Pattern Recognition (CVPR).

[24] Martinelli A. (2012). Vision and IMU Data Fusion: Closed-Form Solutions for Attitude, Speed, Absolute Scale, and Bias Determination. IEEE Trans. Robot..

[25] Weiss S., Scaramuzza D., Siegwart R. (2011). Fusion of IMU and Vision for Absolute Scale Estimation in Monocular SLAM. J. Intell. Robot. Syst..

[26] Wang D., Pan Q., Zhao C., Hu J., Liu L., Tian L. SLAM-based cooperative calibration for optical sensors array with GPS/IMU aided. Proceedings of the International Conference on Unmanned Aircraft Systems.

[27] Shepard D.P., Humphreys T.E. High-precision globally-referenced position and attitude via a fusion of visual SLAM, carrier-phase-based GPS, and inertial measurements. Proceedings of the Position, Location and Navigation Symposium—PLANS 2014.

[28] López E., García A.S., Barea R., Bergasa L.M., Molinos E.J., Arroyo R., Romera E., Pardo S. (2017). A Multi-Sensorial Simultaneous Localization and Mapping (SLAM) System for Low-Cost Micro Aerial Vehicles in GPS-Denied Environments. Sensors.

[29] Zhang J., Singh S., Kantor G. (2012). Robust Monocular Visual Odometry for a Ground Vehicle in Undulating Terrain. Field Serv. Robot..

[30] Valiente D., Gil A., Reinoso Ó., Juliá M., Holloway M. (2017). Improved Omnidirectional Odometry for a View-Based Mapping Approach. Sensors.

[31] Valiente D., Gil A., Payá L., Sebastián J.M., Reinoso Ó. (2017). Robust Visual Localization with Dynamic Uncertainty Management in Omnidirectional SLAM. Appl. Sci..

[32] Engel J., Stückler J., Cremers D. Large-scale direct SLAM with stereo cameras. Proceedings of the IEEE/RSJ International Conference on Intelligent Robots and Systems.

[33] Geiger A., Ziegler J., Stiller C. StereoScan: Dense 3D reconstruction in real-time. Proceedings of the Intelligent Vehicles Symposium.

[34] Tian J.D., Sun J., Tang Y.D. (2011). Short-Baseline Binocular Vision System for a Humanoid Ping-Pong Robot. J. Intell. Robot. Syst..

[35] Zhang L., Shen P., Zhu G., Wei W., Song H. (2015). A Fast Robot Identification and Mapping Algorithm Based on Kinect Sensor. Sensors.

[36] Di K., Qiang Z., Wan W., Wang Y., Gao Y. (2016). RGB-D SLAM Based on Extended Bundle Adjustment with 2D and 3D Information. Sensors.

[37] Henry P., Krainin M., Herbst E., Ren X., Fox D. (2012). RGB-D Mapping: Using Kinect-Style Depth Cameras for Dense 3D Modeling of Indoor Environments. Int. J. Robot. Res..

[38] Endres F., Hess J., Sturm J., Cremers D., Burgard W. (2017). 3-D Mapping With an RGB-D Camera. IEEE Trans. Robot..

[39] Burgard W., Engelhard N., Endres F., Hess J., Sturm J. Real-time 3D visual SLAM with a hand-held RGB-D camera. Proceedings of the RGB-D Workshop on 3D Perception in Robotics at the European Robotics Forum.

[40] Pollefeys M. (2005). Multiple View Geometry. Encyclopedia of Biometrics.

[41] Furukawa Y. (2015). Multi-View Stereo: A Tutorial.

[42] Rublee E., Rabaud V., Konolige K., Bradski G. ORB: An efficient alternative to SIFT or SURF. Proceedings of the International Conference on Computer Vision.

[43] Argyros A. (2004). The Design and Implementation of a Generic Sparse Bundle Adjustment Software Package Based on the LM Algorithm.

[44] Lepetit V., Moreno-Noguer F., Fua P. (2009). EP*n*P: An Accurate O(*n*) Solution to the P*n*P Problem. Int. J. Comput. Vis..

